# Variability in low-flow oxygen delivery by nasal cannula evaluated in neonatal and infant airway replicas

**DOI:** 10.1186/s12931-022-02260-x

**Published:** 2022-12-08

**Authors:** Mozhgan Sabz, Scott Tavernini, Kineshta Pillay, Cole Christianson, Michelle Noga, Warren H. Finlay, Hossein Rouhani, Andrew R. Martin

**Affiliations:** 1 Department of Mechanical Engineering, University of Alberta, Edmonton, AB Canada; 2 Department of Radiology and Diagnostic Imaging, University of Alberta, Edmonton, AB Canada

**Keywords:** Low-flow oxygen delivery, Nasal cannula, In vitro testing, Neonates, Infants, Airway replicas, Fraction of inspired oxygen, Oxygen concentration, Oxygen therapy, Supplemental oxygen

## Abstract

**Background:**

The nasal cannula is considered a trusted and effective means of administering low-flow oxygen and is widely used for neonates and infants requiring oxygen therapy, despite an understanding that oxygen concentrations delivered to patients are variable.

**Methods:**

In the present study, realistic nasal airway replicas derived from medical scans of children less than 3 months old were used to measure the fraction of oxygen inhaled (FiO_2_) through nasal cannulas during low-flow oxygen delivery. Parameters influencing variability in FiO_2_ were evaluated, as was the hypothesis that measured FiO_2_ values could be predicted using a simple, flow-weighted calculation that assumes ideal mixing of oxygen with entrained room air. Tidal breathing through neonatal and infant nasal airway replicas was controlled using a lung simulator. Parameters for nasal cannula oxygen flow rate, nasal airway geometry, tidal volume, respiratory rate, inhalation/exhalation, or I:E ratio (t_i_/t_e_), breath waveform, and cannula prong insertion position were varied to determine their effect on measured FiO_2_. In total, FiO_2_ was measured for 384 different parameter combinations, with each combination repeated in triplicate. Analysis of variance (ANOVA) was used to assess the influence of parameters on measured FiO_2_.

**Results:**

Measured FiO_2_ was not appreciably affected by the breath waveform shape, the replica geometry, or the cannula position but was significantly influenced by the tidal volume, the inhalation time, and the nasal cannula flow rate.

**Conclusions:**

The flow-weighted calculation overpredicted FiO_2_ for measured values above 60%, but an empirical correction to the calculation provided good agreement with measured FiO_2_ across the full range of experimental data.

## Background

Oxygen therapy is used globally to treat hypoxemia in young children with a wide range of respiratory conditions, including pneumonia, bronchiolitis, and acute asthma. Administration of low-flow oxygen through nasal cannulas is the preferred delivery method for children under five years of age who require oxygen therapy [1]. For neonates and infants, use of the nasal cannula is considered a trusted and effective delivery method, despite a widespread understanding that oxygen concentrations delivered to patients are variable [2].

For open patient interfaces such as nasal cannulas, estimating the fraction of inspired oxygen (FiO_2_) delivered to neonates and infants is not straightforward. Variability in FiO_2_ with these devices is likely due, at least in part, to variation in breathing pattern, which influences the entrainment of room air that mixes with supplied oxygen within the upper airway. In the absence of established methods for estimating FiO_2_, clinicians and researchers have extrapolated from studies in adults [3], made rough estimates based solely on oxygen flow rate [4], or simply reported FiO_2_ as indeterminable [5]. Uncertainty in FiO_2_ delivered to young and vulnerable patients has the potential to adversely influence clinical outcomes, given that the consequences of supplying too low, or too high, an oxygen concentration can be severe [6].

Measurement of the FiO_2_ delivered through nasal cannulas to patients is challenging because of the invasiveness of catheters required to sample gas from the hypopharynx [7], or the need to infer FiO_2_ based on non-invasive surrogate measures if catheters are not used [8, 9]. Moreover, simultaneous time-resolved measurements of inhaled oxygen concentration and breathing waveforms are difficult to obtain in vivo in neonates and infants, so previous studies have relied on assumed, rather than measured, breathing parameters when evaluating their influence on FiO_2_ [10]. Nevertheless, previous in vivo studies are unanimous in concluding that FiO_2_ delivered through nasal cannulas to neonates and infants is highly variable across individuals, even at fixed oxygen flow rates [8, 9, 11, 12]. As a consequence, it has been remarked that the FiO_2_ delivered to infants receiving oxygen through nasal cannulas frequently exceeds safe levels [7].

In recent years, in vitro methods incorporating realistic upper airway replicas and breathing simulation have emerged to perform highly controlled and repeatable experiments investigating the performance of devices used in respiratory support [13–21]. In principle, such methods allow unlimited variation of input parameters in experiments conducted within an individual ‘subject,’ or airway replica, enabling detailed assessment of underlying fluid mechanics phenomena influencing, for example, gas concentrations and pressures delivered to the upper airways [20, 22, 23]. Experiments conducted across airway replicas derived from medical scans of different individuals allow the influence of intrasubject variation in upper airway geometry to be assessed [16, 17]. Such in vitro methods complement in vivo investigation by improving the mechanistic understanding of respiratory support across therapies and devices [13, 24], and by providing an advantageous platform for rapid evaluation of early stage prototype devices and patient interfaces [25].

In the present study, we employed realistic upper airway replicas and simulated breathing to evaluate the FiO_2_ delivered to neonates and infants during low-flow oxygen therapy administered through nasal cannulas. Replica geometry, cannula insertion position, tidal volume, respiratory rate, I:E ratio, and oxygen supply flow rate were individually varied to investigate the effect of each parameter on measured FiO_2_. Thereafter, we evaluated the hypothesis that measured FiO_2_ values could be predicted using a simple, flow-weighted calculation that assumes ideal mixing of oxygen with entrained room air.

## Methods

### Nasal airway replicas

Nasal airway replicas were constructed as described in Tavernini et al. [26] using computed tomography (CT) scan data acquired under the condition of anonymity from the University of Alberta Diagnostic Imaging Archives, with approval from the University of Alberta Research Ethics Office. Briefly, the replicas were printed using VeroGray or VeroBlack photopolymers (Stratasys, Eden Prairie, MN, USA) using an Eden 350V system (Stratasys). The replicas were composed of three parts: the first included the face and the nasal vestibule and valve, the second included the turbinate and nasopharynx, and the last extended from the nasopharynx into the trachea. For each airway replica, the outlet was aligned perpendicular to the axis of the trachea and the expected direction of flow. Using this multi-piece construction, support material could be removed after rapid prototyping. Subsequently, the models were sealed with silicone sealant (737, Dow Corning, Midland, MI, USA) and fastened with machine screws. Table 1 shows the demographic and select airway characteristics of the subjects and replicas [26]. Subject 6 was excluded from the study due to the presence of a plastic oral airway that likely changed the shape of the infant’s natural airway and so could not be considered normal. This subject would have been breathing either orally or naso-orally; excluding this subject left a population of only nasal-breathing infants.

### Breathing parameters

**Fig. 1:**
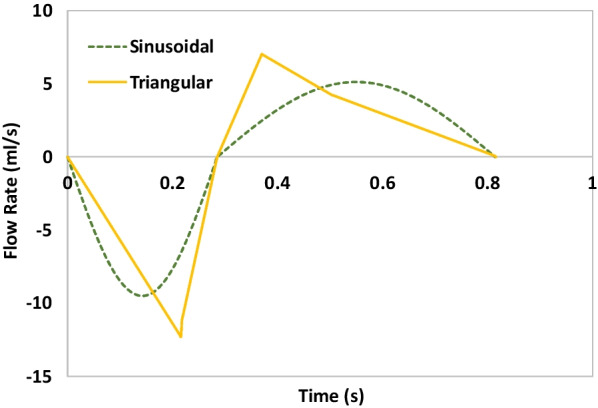
Flow rate is plotted vs. time for a sinusoidal breathing waveform (solid line) with I:E ratio of 3:4 and a triangular waveform (dashed line), approximated from clinical data for an infant with chronic lung disease [27]. Negative flow rates represent inspiratory flow, whereas positive flow rates represent expiratory flow RR = 57, t_i_/t_e_ = 0.6, V_t_ = 28.76 mL)

Simulation of tidal breathing required the following parameters to be specified: respiratory rate (RR), I:E ratio (t_i_/t_e_), tidal volume (V_t_), and the shape of the breathing waveform. Two breathing waveforms were initially examined to evaluate whether the waveform shape significantly influence measured values of FiO_2_. These preliminary measurements were made using two of the seven replicas (subjects 4 and 8). Commonly, prior studies have employed sinusoidal waveforms as an idealized functional form. Here, in addition to a sinusoidal waveform, a triangular waveform, which approximates clinically-observed waveforms for infants with chronic lung disease, was investigated [27]. Figure 1 illustrates both types of waveforms.

A variety of research studies related to chronic lung disease, bronchopulmonary dysplasia, and bronchiolitis were examined to determine an appropriate tidal volume range in neonates and infants with respiratory diseases [27–31]. Based on these publications, representative tidal volumes of 3.5, 5.5, and 7.5 ml/kg were selected for inclusion in the present study. In order to independently assess variability in FiO2 resulting from tidal volume and airway geometry, the average weight of subjects listed in Table 1 (4.89 kg) was used to calculate three fixed tidal volume levels that were then used across all airway replicas. Based on the same set of studies [27–31], the I:E ratio was varied between 1:1 and 3:4, and respiratory rate was varied between 50 and 65 breaths/minute. The selection of the I:E ratios, in particular, was made primarily based on data available for infants with acute bronchiolitis[28]. FiO_2_ was measured in each of the seven replicas for all combinations of tidal volume, respiratory rate, and I:E ratio.

### Nasal cannula flow rate

The flow rate of oxygen provided through nasal cannulas, referred to here as the nasal cannula flow rate, is also an important parameter influencing FiO_2_. As the focus of the present study was low-flow oxygen administration to neonates and infants through nasal cannulas, a limited range of flow rates was investigated. Studies conducted by the National Health Organization and others have determined that the safe range of flow rates for newborns and infants is between 0.5 l/min and 2 l/min [1]. Following clinical guidelines, flow rates of 0.5 l/min and 1 l/min were used for replicas representing subjects less than one month old (subjects 1, 2, 3) or somewhat older (subject 4, 34 days old), whereas values of 1 l/min and 2 l/min were used for replicas representing subjects at least one month old (subjects 5, 7, 8).

### Experimental procedures

**Fig. 2:**
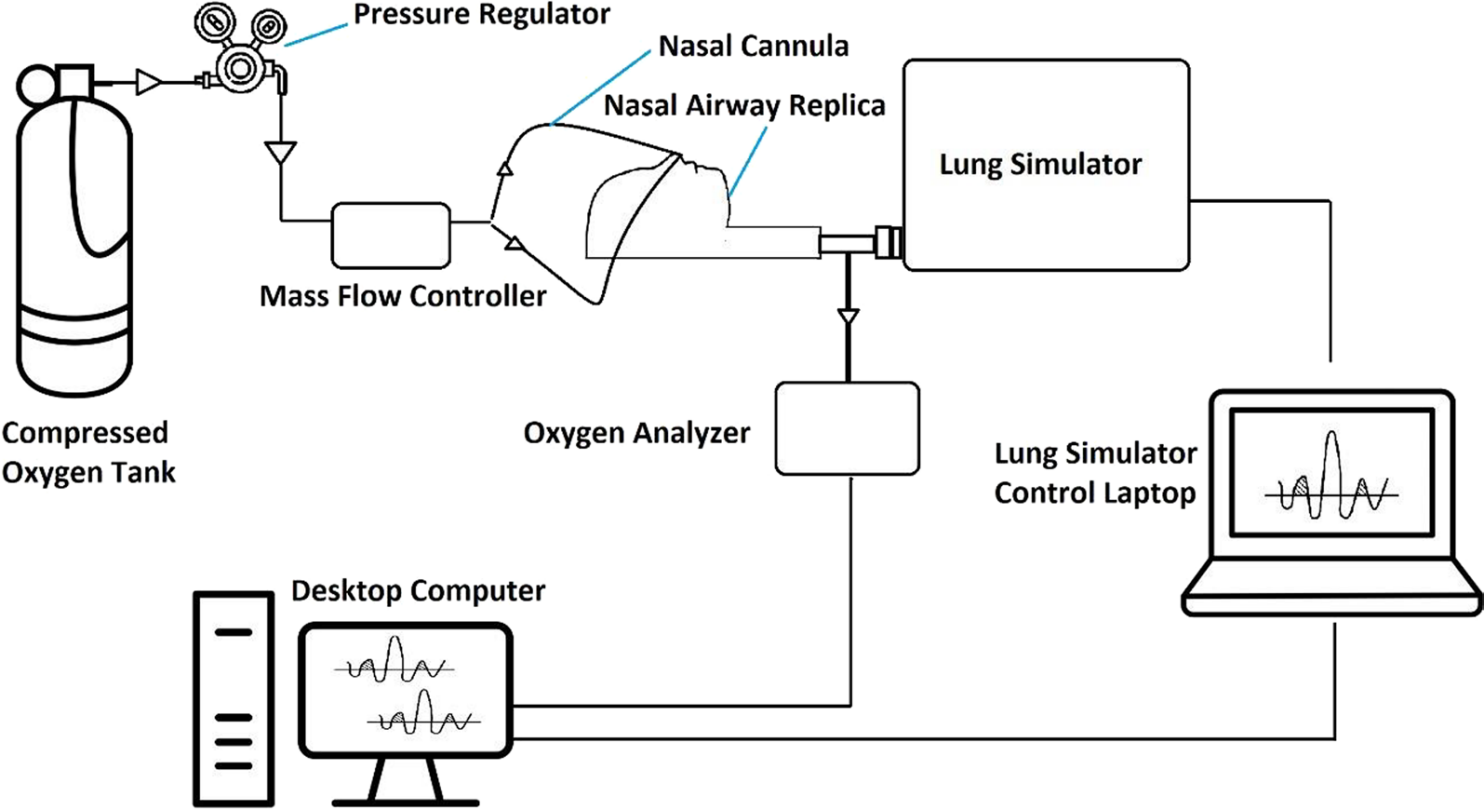
Schematic of the experimental setup. Arrows indicate flow direction

Figure 2 provides a schematic of the experimental setup. A compressed oxygen cylinder (Extra Dry; Linde Canada Inc.) was used as the source of oxygen in this study. A mass flow controller (MCMC-Series Mass Flow Controller; Alicat, USA) was positioned downstream from the cylinder regulator to set the nasal cannula flow rate. A neonatal- or infant-sized nasal cannula (REF: 1611-7-50 or 1601-7-50; Salter Labs, USA), was selected for each replica based on the replica subject age. Experiments were conducted with the cannula prongs fully inserted into the nostrils of the replica and then repeated with cannula prongs inserted approximately halfway (~ 5 mm) into the nostrils. During testing, each replica outlet was in turn connected to a lung simulator (ASL 5000; IngMar Medical, USA) used to mimic the prescribed breathing patterns. The connection tubing had an internal volume of 13.8 ml ± 0.2 ml and included a sampling port for side-stream measurement of the oxygen concentration. Gas was sampled at a flow rate of 200 ml/min, and oxygen concentrations were measured using a fast-response laser diode analyzer (GA-200; iWorx, USA). The oxygen analyzer was calibrated before each series of experiments using a cylinder source of compressed oxygen (99.9% O_2_; Praxair, Canada).

For collection and synchronization, the oxygen concentration-versus time data from the analyzer and the volume-versus-time data from the lung simulator were streamed through an RS-232 serial port and TCP/IP connection, respectively, to a LabVIEW virtual instrument (National Instruments, USA). Volume-versus-time was numerically differentiated to calculate flow rate-versus-time. In each experiment, the flow of oxygen to the nasal cannula was initiated prior to starting the lung simulator in order to produce a characteristic sudden drop in oxygen concentration at the start of the first simulated breath. This signal was used to synchronize concentration and flow-rate data in time. After synchronization, oxygen concentration and flow waveforms were observed to be consistent with expected characteristics [32]; i.e., it was observed that the lowest oxygen fraction corresponded to the highest inhalation flow rate, consistent with expected dilution of oxygen by inhaled room air.

The volume of oxygen inhaled past the trachea during each breath was determined by integrating the product of oxygen concentration and flow rate using the trapezoid rule. Similarly, the inhaled tidal volume was calculated by integrating the flow rate over each inhalation cycle. The FiO_2_ was then determined as the ratio between the volume of inhaled oxygen and the tidal volume. For each experiment, FiO_2_ was averaged over five consecutive breaths after a steady state in expiratory oxygen concentration was observed (after at least 50 breaths). The experiment was repeated in triplicate for each combination of breathing parameters, cannula insertion position, and airway replica.

### Predictive equation

During low-flow oxygen delivery through nasal cannulas, the oxygen concentration in gas inhaled past the upper airway is determined by the mixing of oxygen supplied through the cannula with the entrained room air that forms the bulk of the inhaled breath. As with previous studies evaluating oxygen delivery for infants [10] and adults [18], the average FiO_2_ in the mixed inspiratory flow was estimated using a flow-weighted calculation that assumes ideal mixing of oxygen with entrained room air:

1 $$\frac{{V}_{t}}{{t}_{i}} = {Q}_{i}$$

2 $$Q_{air} = Q_{i} - Q_{NC}$$

3 $${FiO}_{2}\left(\%\right)=\frac{(21{Q}_{air}+100{Q}_{NC})}{({Q}_{air}+{Q}_{NC})}=21+79(\frac{{Q}_{NC}}{{Q}_{i}})$$

Here V_t_ is the inhaled tidal volume, t_i_ is the inhalation time, Q_i_ is the average inhalation flow rate, Q_NC_ is the nasal cannula flow rate, and the room air oxygen concentration is assumed to be 21%.

With reference to the experimental setup (Fig. 2), the FiO_2_ calculated using Eq. (3) was interpreted as the average FiO_2_ exiting nasal airway replicas, approximating an average inhaled oxygen concentration at the trachea.

### Statistical methods

ANOVA procedures employing a set of one-factor repeating measures with Tukey post hoc analysis were used to assess the influence of each parameter on FiO_2_, i.e., replica geometry, nasal cannula flow rate, tidal volume, respiratory rate, I:E ratio, breathing waveform (sinusoidal or triangular), and nasal cannula position (fully- or half-inserted). Results with a two-sided P value less than 0.05 were considered significant.

In addition, the concordance correlation coefficient, ρ_c_, developed by Lawrence and Lin [33], was used to compare predicted and measured FiO_2_ values. This coefficient ranges between 0 and 1, with a higher value indicating better agreement between predicted and measured values.

## Results

### Evaluation of parameters influencing FiO_2_

Preliminary measurements conducted in two replicas (subjects 4 and 8) across the full range of breathing parameters described above revealed no significant influence of the sinusoidal versus triangular waveform on measured FiO_2_ (P = 0.48). Accordingly, all further experiments were conducted using sinusoidal waveforms.

**Fig. 3:**
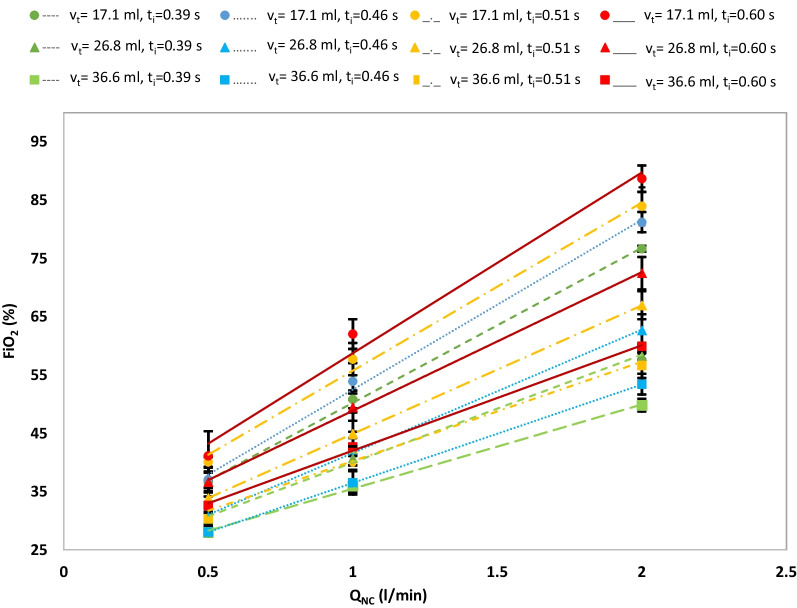
The influence of varying nasal cannula flow rate (Q_NC_), inspiratory time (t_i_) and tidal volume (V_t_) on the fraction of inspired oxygen (FiO_2_) is shown. Similar shapes for data points (circular, triangular, or square shape of points) indicate unique V_t_, and similar line colors and patterns (solid, dashed-dotted, dotted, or dashed lines) indicate unique t_i_. Data points represent average values obtained across replicas, with error bars representing one standard deviation around the average. Data are presented for fully inserted nasal cannula prongs and the sinusoidal breathing pattern

Table 2 provides values of FiO_2_ averaged across the replica geometries with cannula prongs fully inserted into the nares for each combination of tidal volume, respiratory rate, I:E ratio, and nasal cannula flow rate. Across the full parameter range, neither the replica geometry (P = 0.97) nor the cannula position (P = 0.88) influenced measured FiO_2_. Tabulated values of FiO_2_ were instead influenced by the tidal volume, respiratory rate, and nasal cannula flow rate (P < 0.05). At the two levels evaluated, the I:E ratio did not independently influence FiO_2_ (P = 0.42). However, the I:E ratio and the respiratory rate together determined the inhalation time, which influenced FiO_2_ (P = 0.001), as expected from Eqs. 1–3. Figure 3 illustrates the influence of inhalation time, tidal volume, and nasal cannula flow rate on FiO_2_.

**Table 1:** Subject demographics and airway characteristics

		**Demographics**			**Airway characteristics**	
Group	Subject	Age (days)	Sex	Mass (kg)	Volume (mm^3^)	Surface area (mm^2^)
Neonate	1	5	F	3.50	4474	4229
Neonate	2	9	M	3.57	4831	5190
Neonate	3	9	F	3.54	4777	4767
Neonate	4	34	M	N/A	4641	6817
Infant	5	52	F	6.30	4734	4967
Infant	7	78	M	6.60	5752	6855
Infant	8	79	M	5.80	8473	9621
Average (± SD)	–	38	–	4.89 (1.37)	5383 (1318)	6064 (1726)

**Table 2:** Measured FiO_2_ for varying tidal volume (V_t_), respiratory rate (RR), I:E ratio (t_i_/t_e_), and nasal cannula flow rate (Q_NC_)

V_t_ (ml)	RR (/min)	t_i_/t_e_	Q_NC_ (l/min)	FiO_2_ (%)
17.1	65	1:1	0.5	36.9 ± 1.4
17.1	65	1:1	1.0	53.8 ± 3.1
17.1	65	1:1	2.0	81.1 ± 1.7
17.1	65	3:4	0.5	36.5 ± 1.6
17.1	65	3:4	1.0	50.7 ± 1.5
17.1	65	3:4	2.0	76.6 ± 0.5
26.8	65	1:1	0.5	30.6 ± 0.7
26.8	65	1:1	1.0	42.1 ± 2.3
26.8	65	1:1	2.0	62.6 ± 2.7
26.8	65	3:4	0.5	30.2 ± 0.7
26.8	65	3:4	1.0	40.7 ± 2.0
26.8	65	3:4	2.0	58.2 ± 0.7
36.6	65	1:1	0.5	28.0 ± 0.7
36.6	65	1:1	1.0	36.4 ± 1.9
36.6	65	1:1	2.0	53.4 ± 1.8
36.6	65	3:4	0.5	27.9 ± 0.5
36.6	65	3:4	1.0	35.9 ± 1.1
36.6	65	3:4	2.0	49.8 ± 1.0
17.1	50	1:1	0.5	41.0 ± 4.2
17.1	50	1:1	1.0	61.9 ± 2.5
17.1	50	1:1	2.0	88.6 ± 2.7
17.1	50	3:4	0.5	40.0 ± 0.8
17.1	50	3:4	1.0	67.6 ± 2.7
17.1	50	3:4	2.0	83.9 ± 3.2
26.8	50	1:1	0.5	36.5 ± 2.4
26.8	50	1:1	1.0	49.4 ± 2.3
26.8	50	1:1	2.0	72.4 ± 2.7
26.8	50	3:4	0.5	33.8 ± 1.1
26.8	50	3:4	1.0	44.8 ± 3.6
26.8	50	3:4	2.0	66.9 ± 2.4
36.6	50	1:1	0.5	32.5 ± 2.2
36.6	50	1:1	1.0	42.6 ± 1.5
36.6	50	1:1	2.0	59.8 ± 0.3
36.6	50	3:4	0.5	42.3 ± 2.8
36.6	50	3:4	1.0	30.3 ± 1.1
36.6	50	3:4	2.0	56.5 ± 2.1

Values of FiO_2_ are reported as average ± standard deviation. The number of replicas (n) used for calculating average and standard deviation is n = 4 for Q_NC_ = 0.5 l/min, n = 7 for Q_NC_ = 1 l/min, and n = 3 for Q_NC_ = 2 l/min. Data are tabulated for fully inserted nasal cannula prongs the and sinusoidal breathing pattern

### Predicting FiO_2_ values by a flow-weighted calculation

**Fig. 4:**
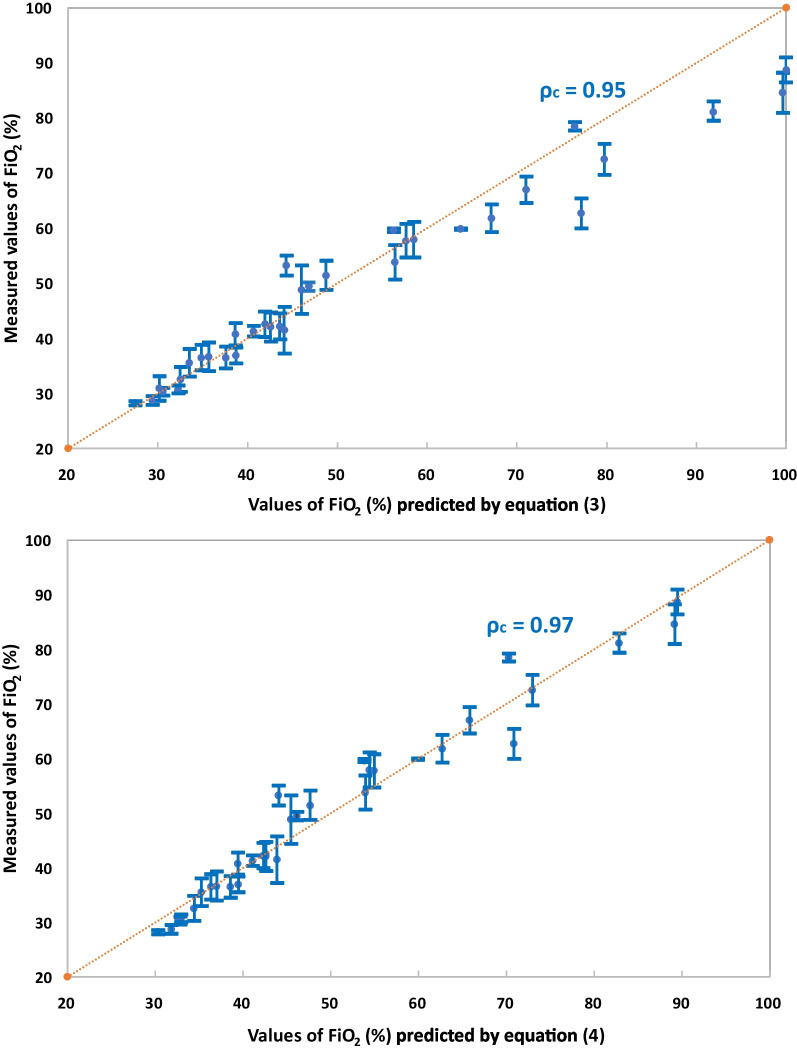
Measured values of FiO_2_ vs. predicted values by **a** Eq. (3) **b** Eq. (4) **c** Eq. (5). Error bars represent one standard deviation around average measured values. ρ_c_ is Lin’s concordance correlation coefficient

FiO_2_ values predicted using Eqs. (1–3) were compared with the measured FiO_2_ values shown in Fig. 4a. While the predictions appeared reasonably consistent with measured data up to FiO_2_ values of approximately 60%, for higher values of FiO_2_ the predictions overestimated the measurements. To improve the predictions for high values of FiO_2_, an empirical correction to Eq. (3) was investigated:

4 $${FiO}_{2}\left(\%\right)=A\left(21+79\left(\frac{{Q}_{NC}}{{Q}_{i}}\right)\right)+B$$

$$A=0.8115$$

$$B=7.9228$$

where the constants *A* and *B* were determined by least squares fitting.

Figure 4b compares FiO_2_ values predicted using Eq. (4) with measured FiO_2_ values. Improvement in the predictions made for high FiO_2_ values is evident, while some overprediction of low measured FiO_2_ values (below 35%) is introduced. Overall, the use of Eq. (4) in place of Eq. (3) resulted in a modest improvement in the concordance correlation coefficient [34, 35].

Finally, to avoid overprediction of measured values at either low or high extremes of FiO_2_, a piecewise approach was investigated, as described by Eq. (5):

5 $$\begin{array}{*{20}c} {FiO_{2} (\% ) = 21 + 79\left( {\frac{{Q_{NC} }}{{Q_{i} }}} \right)} & {\frac{{Q_{NC} }}{{Q_{i} }} < 0.5} \\ {or} & {} \\ {FiO_{2} (\% ) = 0.8115\left( {21 + 79\left( {\frac{{Q_{NC} }}{{Q_{i} }}} \right)} \right) + 7.9228} & {\frac{{Q_{NC} }}{{Q_{i} }} \geq 0.5} \\ \end{array}$$

With reference to Fig. 4c, the use of Eq. (5) provided a further, modest improvement in the concordance correlation coefficient.

## Discussion

The present study evaluated FiO_2_ values delivered to neonates and infants during low-flow oxygen therapy administered through nasal cannulas, using realistic upper airway replicas in combination with simulated tidal breathing. The flow rate of supplied oxygen, in combination with tidal volume and inhalation time, was found to determine FiO_2_. In contrast, neither the nasal airway geometry, nor the position of cannula prongs in the nares (fully inserted versus inserted half-way) influenced measured FiO_2_.

These results help to explain previous observations of high variability in FiO_2_ delivered through nasal cannulas to neonates and infants, even at fixed nasal cannula flow rates [8, 9, 11, 12]. Variability in FiO_2_ was determined by variation in two breathing parameters: tidal volume and inhalation time. Monitoring, or estimating, these parameters is therefore key to improving estimation of FiO_2_ in future research or clinical practice. Accurate estimation of FiO_2_ would be useful when comparing new modes of respiratory support to standard, low-flow oxygen therapy [3]; it might also improve the use of risk prediction tools that include PaO_2_/FiO_2_ or SpO_2_/FiO_2_ ratios for patients receiving low-flow oxygen delivered via nasal cannula [4, 5]. Moreover, improvement in the accuracy of FiO_2_ estimation may help to avoid any deleterious consequences of supplying FiO_2_ outside of safe, target ranges [6, 7].

Based on data obtained using an in vitro methodology like that used in the present study, but with airway replicas and simulated breathing patterns representing adult subjects, Katz et al*.* [18] previously proposed that FiO_2_ may be estimated using Eqs. (1–3), which assume ideal mixing of oxygen supplied through the nasal cannula and entrained room air that forms the bulk of the inhaled breath. The Katz et al*.* formulation is similar to that proposed by Duprez et al*.* [36], who also conducted bench experiments to improve prediction of oxygen delivery to adults, and who also observed a major impact of the average inhalation flow rate on delivered oxygen concentrations. A similar set of algebraic equations was also developed earlier in the context of oxygen delivery to infants by Benaron and Benitz [10], although these authors based the formulation of their equations on inhaled volumes, rather than on average inhaled flow rates. Benaron and Benitz [10] presented predicted values of FiO_2_ that correlated well with hypopharyngeal measurements made in infants receiving oxygen through nasal cannulas, as has been noted by Vain et al. [12]. However, predicted FiO_2_ was observed to consistently overestimate measured concentrations by approximately 4% (absolute % oxygen). The authors attributed differences between predicted and measured values to their use of assumed, rather than measured, values of tidal volume and inhalation time in making their predictions. Benaron and Benitz [10] also noted that the measured hypopharyngeal oxygen concentration may have been lower than predicted because the fraction of mouth breathing was unknown, as their predictions assumed predominantly nasal breathing. The infant population studied by Vain et al. [12] ranged in postnatal age from 19 to 123 days, with an average age of 56 days. While neonates and young infants are commonly considered obligate nasal breathers [37, 38], neither the age at which some infants are able to breathe through their mouths, nor the percentage of infants who do so, has been established [39].

In the present study, which considered simulated nasal breathing through neonatal and infant airway replicas, the ideal mixing calculation summarized in Eqs. (1–3) was found to provide a reasonable prediction of measured FiO_2_ values below approximately 60% (Fig. 4a). Above measured FiO_2_ of 60%, predictions overestimated measured values. From Eq. (3), the limit of 60% FiO_2_ corresponds to a ratio between nasal cannula flow rate and average inhalation flow rate (Q_NC_/Q_i_) of 0.5. Above this value, as the flow rate of oxygen supplied through the nasal cannula approaches the average inhalation flow rate, it is likely that a fraction of supplied oxygen is lost to the room environment during periods of inhalation where the nasal cannula flow rate exceeds the instantaneous inhalation flow rate. Such losses are not accounted for in Eqs. (1–3), which assume ideal mixing of supplied oxygen with entrained room air and would therefore lead to overprediction of FiO_2_. To improve prediction of FiO2 for high flow rate ratios, an empirical correction is proposed here for use when the flow rate ratio, Q_NC_/Q_i_, equals or exceeds 0.5 (Eq. 5). Strong correlation between measured FiO_2_ values and values predicted using Eq. (5) is evident in Fig. 4c.

A limitation of the present study is the use of rigid airway replicas and a lung simulator as opposed to conducting measurements in vivo in human neonates and infants. While use of an in vitro approach permitted controlled and repeatable experiments that would be extremely challenging to conduct in vivo in this age group, this advantage comes with trade-offs. Notably, rigid airway replicas do not deform dynamically with breathing flow rate during the inhalation-exhalation cycle or with administration of small amounts of positive airway pressure. However, the potential influence of dynamic variation in nasal airway geometry on the present results is expected to be insignificant. Such variation is small compared with the intersubject variation in geometry across the seven district airway replicas used in the present study, where replica geometry was found not to significantly influence measured FiO_2_ values. Additionally, while airway replicas do not condition inhaled gases through heat and water transport that occurs in vivo in the nasal airways, differences in gas properties that may influence mixing, such as viscosity and density, between room and body conditions are minor. Finally, experiments conducted using simulated breathing did not include gas exchange, so that gases exhaled back through replicas during tidal breathing remained rich in oxygen and devoid of carbon dioxide. Though the potential for re-inhalation of exhaled gases from room air in the vicinity of the nares has been previously shown to be extremely low [40], gases contained *within* the nasal airway replicas at the end of expiration were inhaled past the oxygen sampling point at the start of the subsequent inhalation cycle. Re-inhalation of oxygen-rich gas from the nasal airway replicas would potentially bias measured FiO_2_ towards higher values than would be expected in vivo [18]. In the present experiments, the magnitude of this effect can be estimated by comparing the average volume of the nasal replicas, 5.38 ml, with inhaled tidal volumes. Assuming in vivo end-expiratory oxygen concentrations 5% below FiO_2_ (absolute %; e.g., for an FiO_2_ of 30%, the end-expiratory oxygen concentration is 25%), the omission of gas exchange is expected to bias measured FiO_2_ upwards on average by 1.6% (absolute) at the lowest tidal volume studied (17.1 ml). At the highest tidal volume studied, 36.6 ml, this upwards bias is reduced to 0.7%. In either case, the change in measured FiO_2_ owing to gas exchange is smaller than the precision of the repeated experiments and not anticipated to be clinically important.

## Conclusions

Realistic nasal airway replicas and simulated breathing patterns were used to evaluate the FiO_2_ delivered to neonates and infants during low-flow oxygen therapy administered through nasal cannulas. The flow rate of supplied oxygen, in combination with tidal volume and inhalation time, was found to determine FiO_2_. Neither the nasal airway geometry, nor the position of cannula prongs in the nares (fully inserted versus inserted half-way) influenced measured FiO_2_. An empirical correlation was presented that provides good agreement with measured FiO_2_ across the full range of experimental data, from 27.9 to 88.6%. Improved prediction of FiO_2_ delivered to neonates and infants receiving standard, low-flow oxygen therapy is expected to be of value in maintaining oxygenation within safe, target ranges, and may contribute to increased use of risk prediction ratios involving FiO_2_.

## Data Availability

All supporting data is available on request.
