# Correction to: Variability in low-flow oxygen delivery by nasal cannula evaluated in neonatal and infant airway replicas

**DOI:** 10.1186/s12931-023-02379-5

**Published:** 2023-03-27

**Authors:** Mozhgan Sabz, Scott Tavernini, Kineshta Pillay, Cole Christianson, Michelle Noga, Warren H. Finlay, Hossein Rouhani, Andrew R. Martin

**Affiliations:** 1 Department of Mechanical Engineering, University of Alberta, Edmonton, AB Canada; 2 Department of Radiology and Diagnostic Imaging, University of Alberta, Edmonton, AB Canada

**Correction to: Respiratory Research (2022) 23: 333** 10.1186/s12931-022-02260-x

Following publication of the original article [1], the authors identified that Fig. 4c in Fig. 4 was missing. It has been updated in the correction. The correct figure is given below.
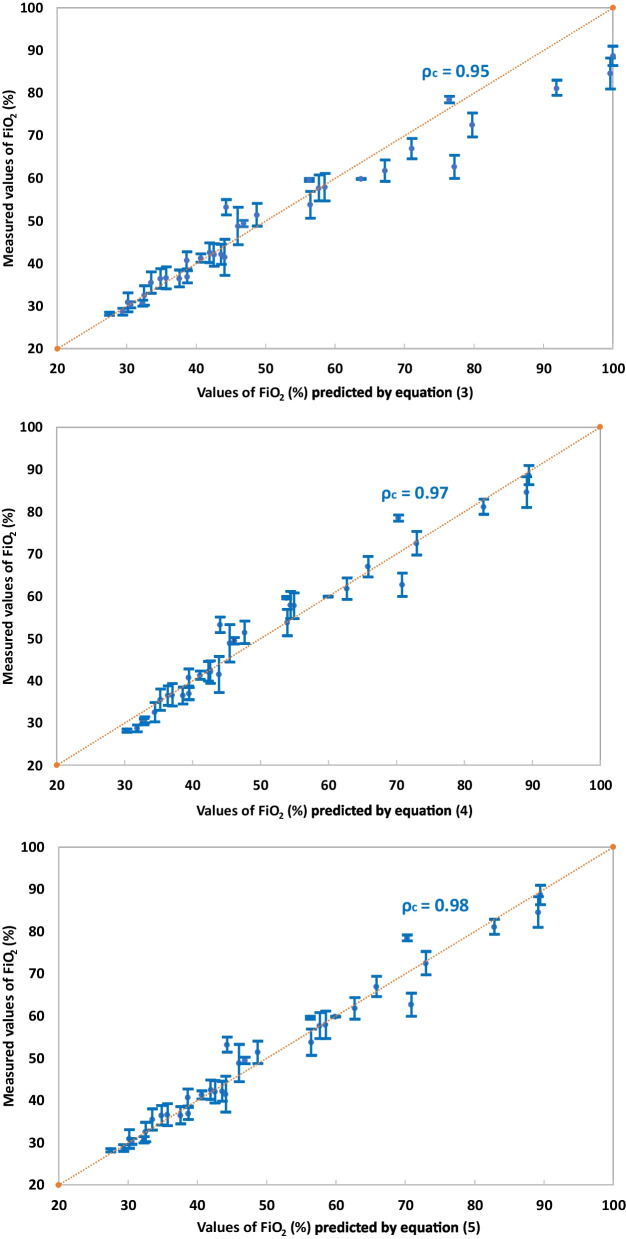


The Figure 1 caption has been corrected.

Fig. 1 Flow rate is plotted vs. time for a sinusoidal breathing waveform (dashed line) with I:E ratio of 3:4 and a triangular waveform (solid line), approximated from clinical data for an infant with chronic lung disease [27]. Negative flow rates represent inspiratory flow, whereas positive flow rates represent expiratory flow RR = 57, ti/te = 0.6, Vt = 28.76 mL)

The original article has been updated
